# Improved Cd, Zn and Mn tolerance and reduced Cd accumulation in grains with wheat-based cell number regulator TaCNR2

**DOI:** 10.1038/s41598-018-37352-6

**Published:** 2019-01-29

**Authors:** Kun Qiao, Fanhong Wang, Shuang Liang, Hong Wang, Zhangli Hu, Tuanyao Chai

**Affiliations:** 1 College of Life Science, University of Chinese Academy of Sciences, Beijing, China; 2 Shenzhen Key Laboratory of Marine Bioresource & Eco-environmental Science, Guangdong Engineering Research Center for Marine Algal Biotechnology, College of Life Science and Oceanography, Shenzhen University, Shenzhen, China; 3 Southeast Asia Biodiversity Research Institute, Chinese Academy of Science, Yezin, Nay Pyi Taw 05282 Myanmar; 4 The Innovative Academy of Seed Design (INASEED), Chinese Academy of Sciences, Beijing, China

## Abstract

Soil microelement deficiency and heavy metal contamination affects plant growth and development, but improving trace element uptake and reducing heavy metal accumulation by genetic breeding can help alleviate this. Cell number regulator 2 (TaCNR2) from common wheat (*Triticum aestivum*) are similar to plant cadmium resistance proteins, involved with regulating heavy metal translocation. Our aim was to understand the effect of TaCNR2 on heavy metal tolerance and translocation. In this study, real-time quantitative PCR indicated *TaCNR2* expression in the wheat seedlings increased under Cd, Zn and Mn treatment. Overexpression of *TaCNR2* in *Arabidopsis* and rice enhanced its stress tolerance to Cd, Zn and Mn, and overexpression in rice improved Cd, Zn and Mn translocation from roots to shoots. The grain husks in overexpressed rice had higher Cd, Zn and Mn concentrations, but the brown rice accumulated less Cd but higher Mn than wild rice. The results showed that TaCNR2 can transport heavy metal ions. Thus, this study provides a novel gene resource for increasing nutrition uptake and reducing toxic metal accumulation in crops.

## Introduction

With increased global industrialization, there is greater discharge of garbage and wastewater with the result that excessive toxic heavy metals accumulate in soil. Heavy metals, such as zinc (Zn), manganese (Mn), iron (Fe), copper (Cu) and cobalt (Co) are essential microelements^1^. Zn and Mn are cofactors of many enzymes, participate in the synthesis of proteins and carbohydrates, and regulate photosynthesis^2–4^, but an excess or deficiency of either can affect plant growth. An increase in Zn concentrations results in significantly inhibited seedling growth, and decreased biomass accumulation, plant height and leaf area^5,6^. Similarly, Zn deficiency also causes plants to grow slowly as a result of reduced photosynthesis rates^7^. Both high Mn and a deficiency will significantly increase the permeability of a cell’s plasma membrane and greatly reduce peroxidase and catalase activity^8^. The enzyme activity of ascorbic acid and glutathione reductase was shown to increase under high Mn stress in cucumbers^9^.

In addition, cadmium (Cd), lead (Pb), mercury (Hg) and arsenic (As) are non-essential elements and affect plant growth. Cd is one of the most toxic pollutants in soil^10,11^, and it mainly derives from sewage sludge disposal, pesticides, fungicides and phosphorus-rich fertilizer use, such as industrial and agricultural production. Cd can seriously destroy the plant roots, as well as affect the normal growth and development of plants^12,13^. Furthermore, Cd easily accumulates in crops, and in this way, can also enter the food chain and threaten human health^14,15^. Cd strongly inhibits many enzyme activities and can thus affect the enzymatic system of the liver, lungs and spleen. Cd causes kidney malformation or malfunction, hinders skeletal development, and affects the reproductive system^16^. Therefore, the key to improving crop quality and food safety is to maintain the ion balance of trace elements in crops and reduce the accumulation of toxic heavy metals.

Recently, trace element deficiencies and accumulation of toxic metals in plants had attracted attention from researchers. To reduce the concentrations of heavy metals in crops, a number of remediation techniques in soil have been used. The advantages of bioremediation include its low cost, lack of secondary pollution and the fact that it does not change the nature of the soil. However, it takes time and is laborious, and, most importantly, it does not fundamentally reduce the heavy metal concentrations in crops.

At present, heavy metal transporters have been used in plants to improve their ion balance. As a result, an increasing number of heavy metal transporters have been isolated and studied. Metal-tolerance proteins, members of the cation diffusion facilitator family, are highly specific for transporting Zn, but they can also transport Co^2+^, Fe^2+^ and Cd^2+^ ^17–19^. Heavy metal ATPase can transport heavy metals across membranes^11,14,20^, and plays an important role in transporting Zn/Cd from plant roots to shoots. The natural resistance-associated macrophage protein is a major Mn transporter^21^ and also participates in Fe^2+^, Zn^2+^ and Cd^2+^ transportation^22–24^. Plant cadmium resistance proteins (PCR) are involved with transporting Zn^2+^ and Cd^2+^ and in *Arabidopsis* one PCR, AtPCR1, a Cd-efflux transporter, improves the tolerance of Cd by exporting Cd out of the cell and decreasing its concentration^25^. Additionally, AtPCR2 is a Zn-efflux transporter, which can regulate Zn redistribution in plants^26^. However, the use of these transporters cannot meet the current demand for heavy metal treatments.

Previously, fruit-weight 2.2 (FW2.2) from tomato was located through quantitative trait locus, and regulated plant growth and development^27^. Maize cell number regulator 1 (ZmCNR1), a maize homolog of FW2.2, decreased fruit weight of transgenic maize^28^. Two of them were reported to regulate cell numbers and organ size^27,28^. It was found that the sequence of CNR and PCR were highly similar, as they both contain placenta-specific 8 (PLAC8) domains^29^. Furthermore, CNR and PCR both contained the CC/LXXXXCPC conserved motif. However, a specific CNR, ZmCNR2 from *Zea mays*, had been suggested to be involved in Cd tolerance and chelation^28^. Therefore, we wanted to investigate whether other CNRs could regulate heavy metal transport. In this study, a *CNR2* from common wheat (*Triticum aestivum*) was isolated (*TaCNR2*), and its expression was analyzed under Cd, Zn and Mn stresses by real-time quantitative PCR. The overexpression of *TaCNR2* in yeast, *Arabidopsis* and rice was used to determine stress tolerance to Cd, Zn and Mn. Heavy metal content was measured in the seedlings of overexpressed *Arabidopsis* and rice, grains of brown rice and husks of mature rice. This study identifies a potential transporter for cultivation by genetic breeding, which is useful for improving crop yields and food security, and managing heavy metal contamination in soil.

## Results

### Identification and characterization of *TaCNR2*

**Figure 1:**
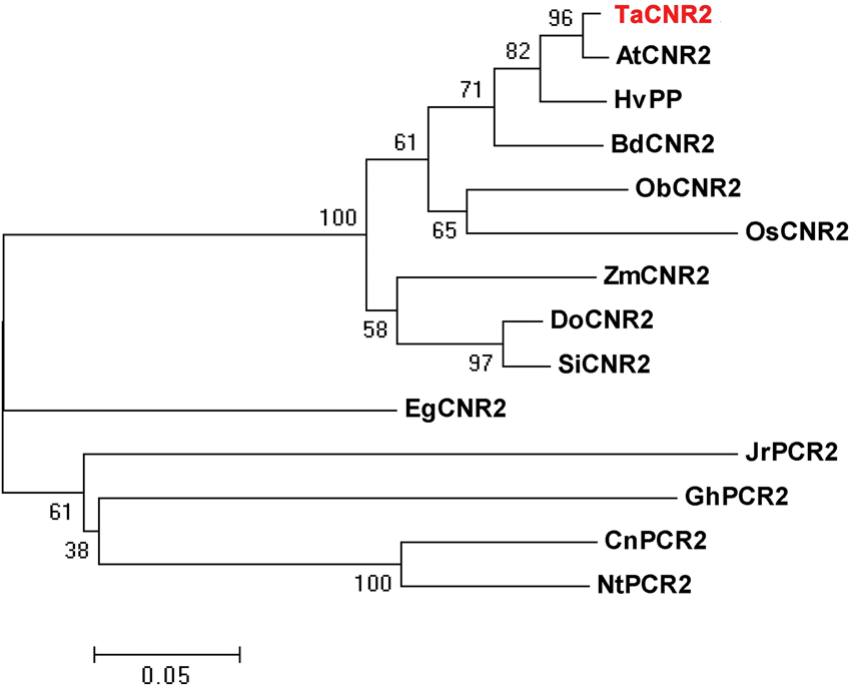
The neighbor-joining (NJ) phylogenetic relationship of *TaCNR2* proteins with CRN2 and PCR2 from other species. CRN2: Ta, *Triticum aestivum*; At, *Aegilops tauschii*; Hv, *Hordeum vulgare*; Si, *Setaria italic*; Zm, *Zea mays*; Os, *Oryza sativa*; Ob, *Oryza brachyantha*; Bd, *Brachypodium distachyon*; Do, *Dichanthelium oligosanthes*; Eg, *Elaeis guineensis*. PCR2: Nt, *Nicotiana tomentosiformis*; Gh, *Gossypium hirsutum*; Ca, *Capsicum annuum*; Jr, *Juglans regia*. TaCNR2 was marked in red.

**Figure 2:**
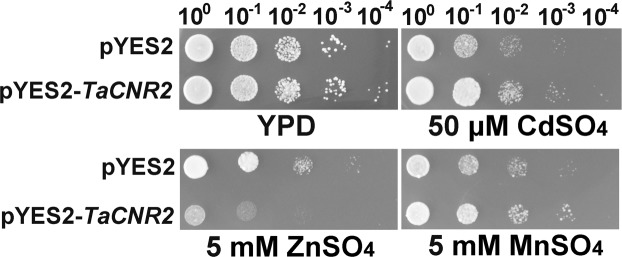
*TaCNR2*-transgenic yeast grown under heavy metal treatments. The pYES2 and pYES2-*TaCNR2* were transformed into yeast strain (BY4741). Gradient dilution was spotted onto the solid media (yeast extract; peptone; galactose) with 50 μM CdSO_4_, 5 mM ZnSO_4_ and 5 mM MnSO_4_. The YPD medium (yeast extract; peptone; glucose) was the control. Growth was maintained at 30 °C for 3–7 days.

The *TaCNR2* gene was isolated from common wheat (*Triticum aestivum*), and the evolutionary relationship showed that it clustered with the CNR2 of *Aegilops tauschii*, *Hordeum vulgare* and *Brachypodium distachyon*, then clustered with the PCR2 of other species (Fig. 1). Therefore, the gene was named *TaCNR2*. In the yeast tolerance assay, *TaCNR2* transgenic lines had better growth than the control pYES2 at 50 μM CdSO_4_ and 5 mM MnSO_4_, but the growth was distinctly weaker than pYES2 under 200 μM ZnSO_4_ (Fig. 2).

### Expression characteristics of *TaCNR2*

**Figure 3:**
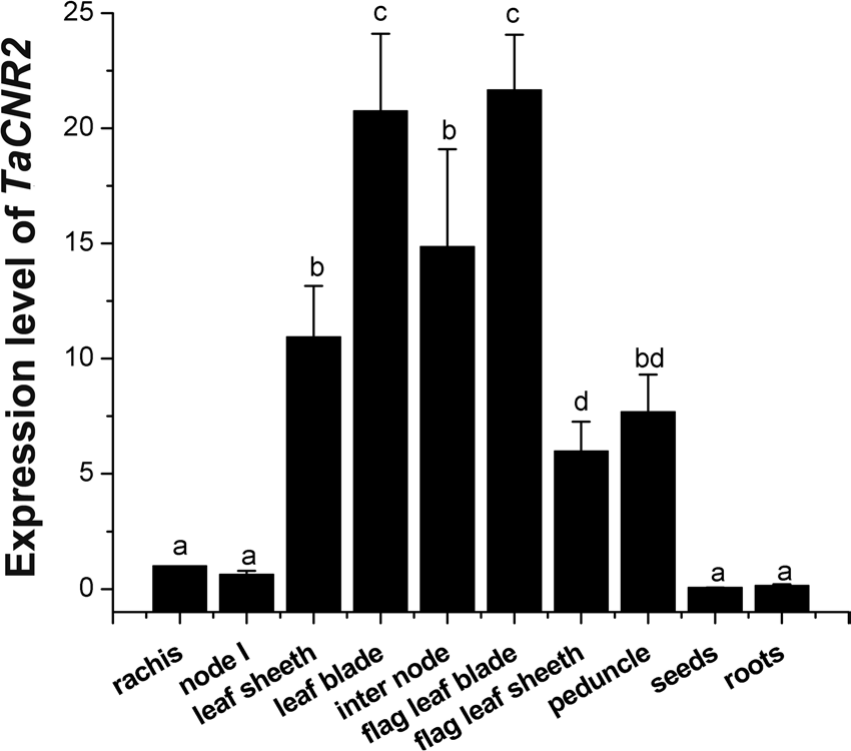
The expression analysis of *TaCNR2* in different wheat tissues including roots, internodes, node I, leaf sheaths, leaf blades, flag leaf sheaths, flag leaf blades, peduncles, rachises and seeds. Values show the mean ± SE of three independent experiments. One-way ANOVA was used to analyze the data and is represented by letters (*p* < 0.05; **a**–**d**).

**Figure 4:**
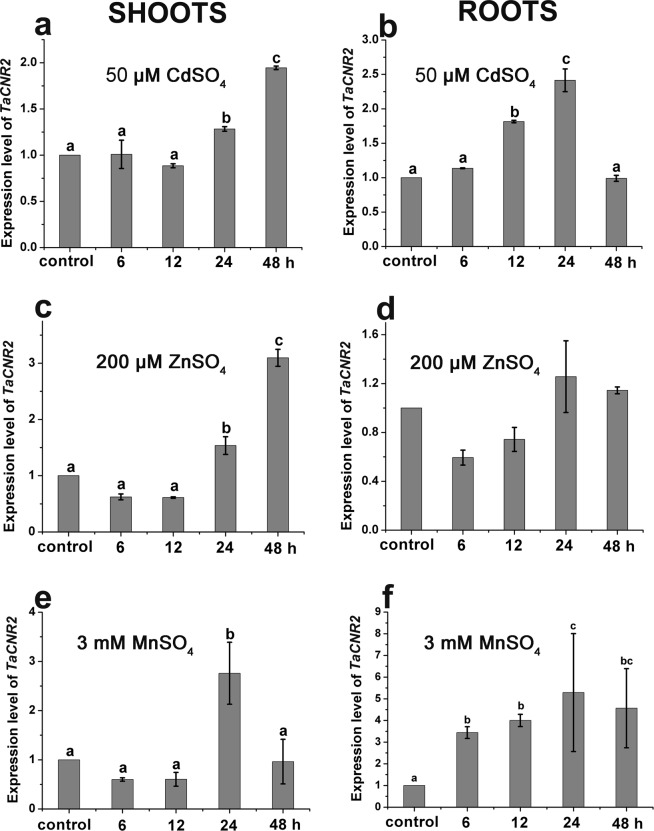
The *TaCNR2* relative expression in the shoots and roots of wheat seedlings under 50 μM CdSO_4_ (**a,b**), 200 μM ZnSO_4_ (**c,d**) and 3 mM MnSO_4_ (**e,f**). The treatment time was 0, 6, 12, 24 and 48 h. Values show the mean ± SE of three independent experiments. One-way ANOVA was used to analyze the data and is represented by letters (*p* < 0.05; **a**–**d**).

The expression of *TaCNR2* in different wheat tissues was determined. The leaf blade and flag leaf blade had the maximum expression of all wheat tissues, about 20-fold higher than the rachis, while the internode also had 14-fold higher expression than the rachis (Fig. 3). To verify whether *TaCNR2* was induced by heavy metals, the expression was tested under Cd, Zn and Mn treatment. The untreated wheat seedlings served as control. Under 50 μM CdSO_4_, the seedling shoots had slightly higher expression than the control at 24 and 48 h, while the expression of roots was higher than the control at 12 and 24 h (*p* < 0.05, Fig. 4a,b). Expression of *TaCNR2* in the shoots was 3-fold higher than the control at 48 h (*p* < 0.05, Fig. 4c), but the expression was no different in roots treated with 200 μM ZnSO_4_ (Fig. 4d). The expression of *TaCNR2* in the shoots did not change after 6 and 12 h, after they increased and reached a maximum at 24 h under 3 mM MnSO_4_ (*p* < 0.05, Fig. 4e). In the roots, *TaCNR2* expression increased at 6 h, with the highest expression at 24 h, about 5-fold higher than the control (*p* < 0.05, Fig. 4f).

### TaCNR2 enhanced the tolerance of Cd, Zn and Mn

**Figure 5:**
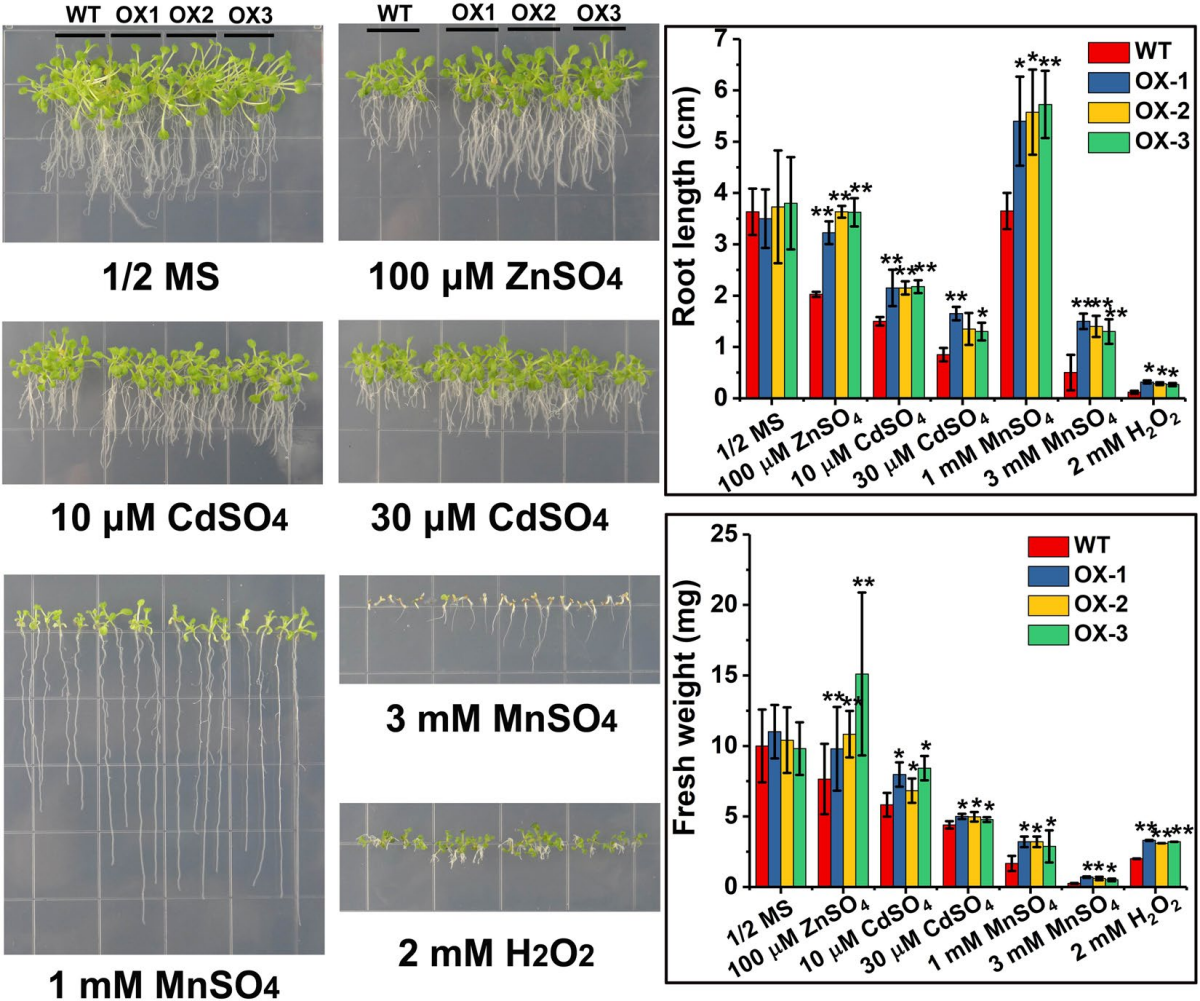
The stress tolerance of *TaCNR2*-transgenic *Arabidopsis* after treatment with 100 μM ZnSO_4_, 10 and 30 μM CdSO_4_, 1 and 3 mM MnSO_4_, and 2 mM H_2_O_2_. Values are presented as the mean ± SE of three independent experiments. The root length and fresh weight were measured. The asterisks show obvious differences between WT and three overexpression lines (*0.01 < *p* < 0.05).

To understand TaCNR2 tolerance to heavy metals, the length and fresh weight of overexpressed *TaCNR2 Arabidopsis* and rice was determined. The three *TaCNR2*-transgenic *Arabidopsis* (OX-1, OX-2 and OX-3) and WT had similar growth under 1/2 MS solid media without heavy metal treatment. However, the root length and fresh weight of the overexpressed lines were distinctly better than WT under 100 μM ZnSO_4_, 10 and 30 μM CdSO_4_, 1 and 3 mM MnSO_4_ and 2 mM H_2_O_2_ (*p* < 0.05, Fig. 5).

**Figure 6:**
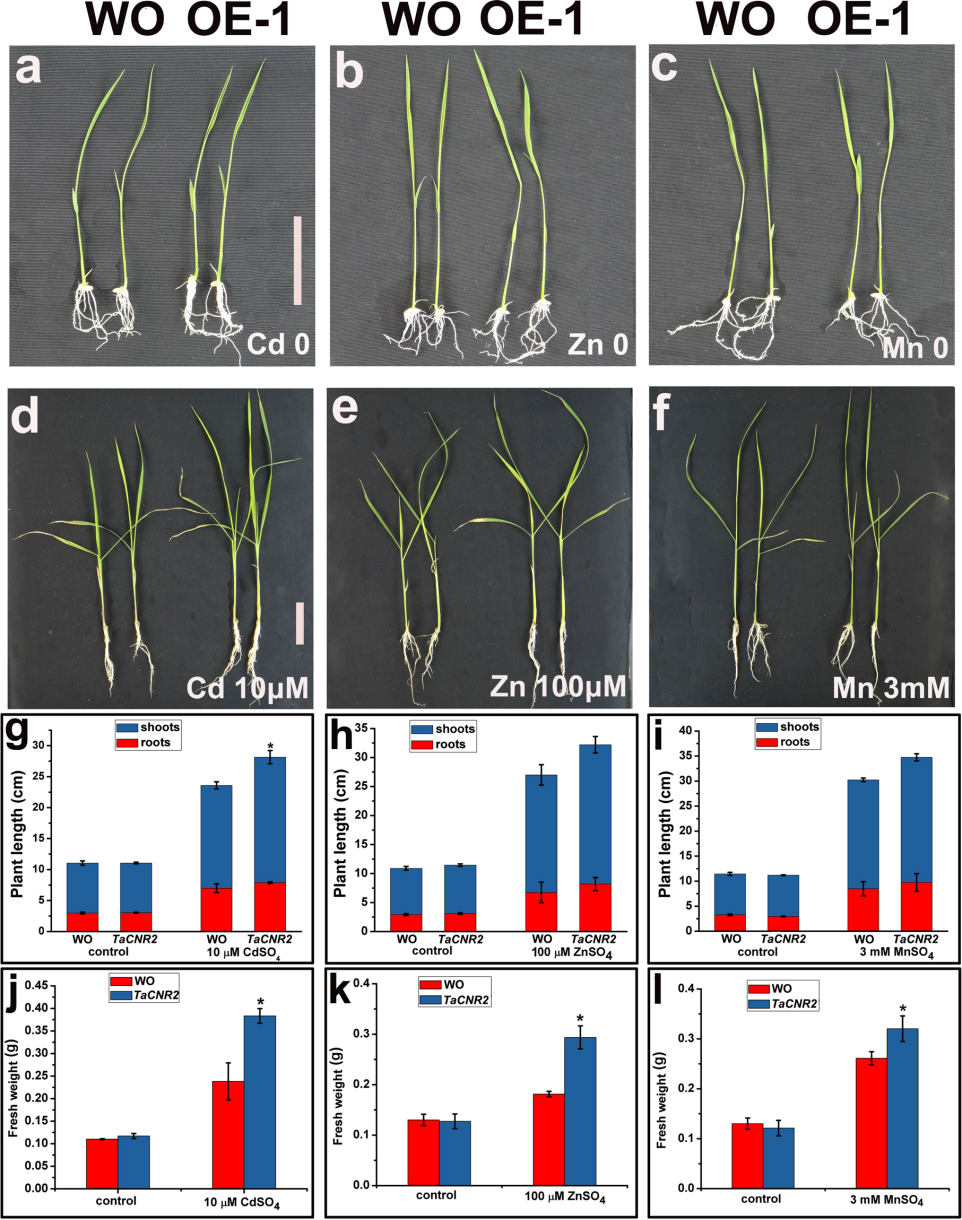
The tolerance analysis of overexpressed *TaCNR2* in rice after treatment with 10 μM CdSO_4_, 100 μM ZnSO_4_ and 3 mM MnSO_4_. Values are presented as the mean ± SE of three independent experiments. The root length and fresh weight were measured. The asterisks show obvious differences between WO and overexpression lines (OE-1) (*0.01 < *p* < 0.05).

The growth of overexpressed *TaCNR2* rice was no different with wild-type rice (WO) in 1/2 HS liquid media without exposure to Cd, Zn and Mn stress (Fig. 6a–c). In samples treated with 30 μM CdSO_4_, the shoot length and fresh weight of transgenic lines (OE-1) were better than WO (*p* < 0.05, Fig. 6d,g,j). The shoot length of overexpressed lines was slightly higher than WT (Fig. 6e,f,h,i), and the fresh weight was significantly higher than WO at 100 μM ZnSO_4_ and 3 mM MnSO_4_ (*p* < 0.05, Fig. 6k,i).

### TaCNR2 improved Cd, Zn and Mn translocation

**Figure 7:**
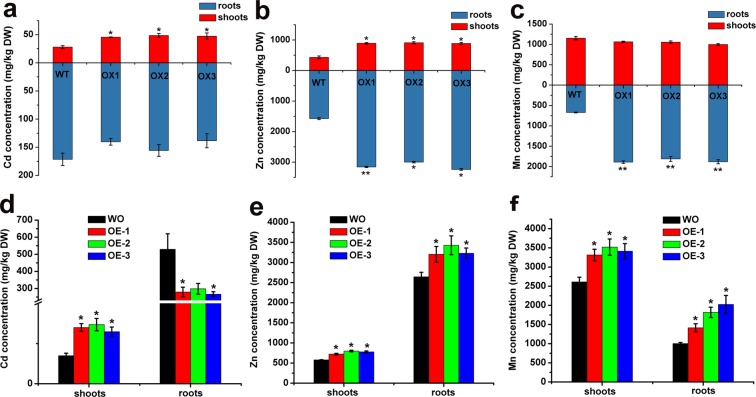
Heavy metal concentrations in overexpressed *TaCNR2* in *Arabidopsis* and rice after CdSO_4_, ZnSO_4_ and MnSO_4_ treatments. The heavy metal content of shoots and roots were measured by ICP-OES. Values are presented as the mean ± SE of three independent specimens. The asterisks indicate significant differences between WS and overexpressors (*0.01 < *p* < 0.05).

To understand the heavy metal transport influence of TaCNR2, the metal ion content of the seedlings of transgenic *Arabidopsis* and rice was determined after treatment with Cd, Zn and Mn. The three overexpressed *Arabidopsis* lines (OX-1, OX-2 and OX-3) had higher Cd and Zn concentrations in the shoots than WT at 30 μM CdSO_4_ and 200 μM ZnSO_4_ (*p* < 0.05, Fig. 7a,b). However, the Mn shoot content in overexpressing lines was not significantly different from WT, but over-accumulated in the roots (Fig. 7c).

In addition, overexpression of *TaCNR2* in the shoots of rice resulted in distinctly higher Cd, Zn and Mn concentrations than WO under 30 μM CdSO_4_ and, 200 μM ZnSO_4_ and 3 mM MnSO_4_, respectively. The Cd content of the roots was lower than WO, but Zn and Mn were higher than in WO (*p* < 0.05, Fig. 7d–f).

### TaCNR2 reduced Cd accumulation in grains of brown rice

**Figure 8:**
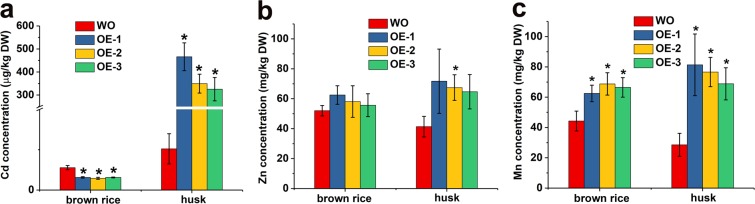
The Cd, Zn and Mn content of *TaCNR2*-transgenic rice in brown rice and grain husks after CdSO_4_, ZnSO_4_ and MnSO_4_ treatments. Values are presented as the mean ± SE of three independent specimens. The asterisks indicate significant differences between WS and overexpressors (*0.01 < *p* < 0.05).

To understand the influence of TaCNR2 on the accumulation of heavy metals, Cd, Zn and Mn content of brown rice and husks of overexpressed mature rice were measured. The husk Cd, Zn and Mn contents in overexpressed lines were all higher than WO (*p* < 0.05, Fig. 8a–c). The Mn content of overexpressed lines were obviously higher than WO in brown rice (*p* < 0.05, Fig. 8c). However, Zn content in brown rice from transgenic lines was no different than WO (Fig. 8b), and had lower Cd concentrations (*p* < 0.05, Fig. 8a).

## Discussion

Common wheat is an important staple food for humans and is one of the three major cultivated grains. In 2013, heavy metals were detected in wheat from more than 20 provinces in China, and Cd concentrations exceeded the national food standards by >20 times. An important source of these heavy metals is contaminated soil. Hence, preventing and controlling heavy metal pollution in crops is an urgent and difficult challenge. The Chinese spring wheat is a very important variety of common wheat, and is widely used in wheat genetic research.

Although TaCNR2 from *Triticum aestivum* has a closely evolved relationship with CNR2, it also has a high similarity to PCR2 (Fig. 1). Furthermore, TaCNR2 has the same AtPCR1, AtPCR2 and OsPCR1 containing CCXXXXCPC motif (data not shown), which was reported to be involved in Zn and Cd tolerance and transportation^25,26,30^.

The maximum expression of *TaCNR2* in different wheat tissues was found in the leaf blade, flag leaf blade and internode. The results suggested that TaCNR2 may participate in the transportation of water and inorganic salts from the internode to the leaf. The expression level was induced to increase in the wheat seedlings under Cd, Zn and Mn (Fig. 2), indicating TaCNR2 may be involved in the binding and transport of heavy metals. In our experiments, genetically modified yeast, *Arabidopsis* and rice were used to analyze TaCNR2 function. Yeast with *TaCNR2* were more sensitive to Zn stress, which may cause excess Zn to be absorbed in the cells, resulting in zinc poisoning that leads to weak growth. However, TaCNR2 may prevent the uptake of Cd and Mn, or export Cd and Mn out of the cell, which would reduce the concentration of those heavy metals within the cell. *Arabidopsis* AtPCR1 can enhance Cd tolerance in yeast, which reduces the Cd concentration in yeast cells and decreases Cd toxicity^25^.

Overexpressed *TaCNR2 Arabidopsis* and rice both had tolerance to Cd, Zn and Mn, and enhanced Cd and Zn translocation from roots to shoots, but Mn translocation was prevented in *Arabidopsis* (Figs 5–7), suggesting that TaCNR2 may enhance the translocation of Cd, Zn, and Mn to tolerate the stress of heavy metals. Overexpression of *AtPCR1* also improved the tolerance to Cd, and removed Cd from *Arabidopsis* protoplast^25^. AtPCR2 had strong tolerance to Cd and Zn, and the mutant *atpcr2* was more sensitive to Cd and Zn than WT. The Zn concentration in overexpressed *AtPCR2 Arabidopsis* roots was significantly lower than WT, suggesting AtPCR2 decreased Zn toxicity by excreting it from the roots^26^. The Cd and Zn tolerance in overexpressed *TaCNR2 Arabidopsis* and rice was stronger than WT (Figs 5 and 6), a similar result to AtPCR2. However, Zn concentrations in overexpressed *TaCNR2 Arabidopsis* and rice shoots and roots were higher than WT; presumably, excess Zn was transported to the shoots to relieve the Zn concentration in the roots. Overexpression of *TaCNR2* in *Arabidopsis* led to better Mn tolerance, but not Mn translocation, suggesting excess Mn was not transported to the shoots, which reduced Mn toxicity. The growth of overexpressed *TaCNR2* rice was slightly better than WO when treated with Mn, and the shoots and roots had more Mn than WO (Fig. 7). The results indicated that excess Mn was transported into the shoots, and may inhibit rice growth.

The Zn content of brown rice and grain husks in *OsPCR1* knockout mutants was higher than WT rice^30^. In our study, Cd, Zn and Mn content in husks of *TaCNR2*-transgenic rice were all higher than WO, while these heavy metals in brown rice of overexpressed *TaCNR2* lines had lower Cd content, but Zn concentrations showed no distinction from WO (Fig. 8); however, the Mn concentrations were higher than WO, illustrating that the metal ion supply to the brown rice and husks is through two different transport channels. These indicated that TaCNR2 was involved in the transport of heavy metals (Cd, Zn and Mn) to grains, but it hindered the translocation and accumulation of Cd in brown rice.

The tolerance to Cd, Zn and Mn in *TaCNR2*-transgenic yeast, *Arabidopsis* and rice slightly differed, and the translocation of Cd, Zn and Mn in transgenic *Arabidopsis* and rice was similar, suggesting TaCNR2 had different tolerances and transport mechanisms in different organisms. However, the *TaCNR2* in transgenic yeast, *Arabidopsis* and rice enhance Cd tolerance and translocation from roots to shoots.

In summary, the expression of *TaCNR2* increased under Cd, Zn and Mn treatments. *TaCNR2* overexpressed in *Arabidopsis* and rice exhibited Cd, Zn and Mn tolerance, and strong translocation of Cd, Zn and Mn in rice. TaCNR2 can reduce the Cd accumulation in brown rice, and enhance Mn content in husks. The results of this study suggest a feasible heavy metal transporter, and could play an important role in maintaining the ion balance of plants. These aspects could help us improve crop yield and quality, and maintain food security.

## Materials and Methods

### Plant conditions and treatment

Seeds of the common wheat *Triticum aestivum* were germinated in sterile deionized H_2_O on glass plates for three days, then transferred to 1/2 Hoagland’s solution (HS, pH 5.8) for five days^31^. The culture conditions were 22 °C under a photoperiod of 8:16 h light: dark. After six days, the seedlings were transferred to 1/2 HS fluid media supplemented with 50 μM CdSO_4_, 200 μM ZnSO_4_ and 3 mM MnSO_4_. The shoot and root samples were separated and collected after 0, 6, 12, 24 and 48 h. The seedlings were vernalized at 4 °C for 10 days and cultivated in agricultural soil for four months to obtain different wheat tissues, including roots, internodes, node I, leaf sheaths, leaf blades, flag leaf sheaths, flag leaf blades, peduncles, rachises and seeds. All samples were ground into powder after application of liquid nitrogen. All primer sequences used for the PCR reactions are listed in Table 1.

**Table 1:** List of primer sequences used in this study.

Primer	Sequence(5′-3′)
TaCNR2-F	ATGTACCCGAAGCCGGAGG
TaCNR2-R	TCAACGGGTCATCCCTGGGTGC
BXTaCNR2-F	GGATCCATGTACCCGAAGCCGGAGG
BXTaCNR2-R	TCTAGATCAACGGGTCATCCCTGGGTGC
Taactin-F	CTTTCACAACCACTGCCGAGC
Taactin-R	CACTTGACCATCAGGCAGCTC
TaCNR2-FSSDL	AGGTGGCGGAGATCATCGACC
TaCNR2-RSSDL	TTGAGCTCGCGGTACTCCTGG
TaCNR2-BFW	GGATCCATGTACCCGAAGCCGGAGGATG
TaCNR2-xhRV	CTCGAGTCAACGGGTCATCCCTGGGTG
TaCNR2-1301-FW	CAGGTCGACTCTAGAGGATCCATGTACCCGAAGCCGGAGGATG
TaCNR2-1301-RV	GAGCTCGGTACCCGGGGATCCTCAACGGGTCATCCCTGGGTGC

### Gene cloning

Total RNA was isolated from 6 day old wheat seedlings using RNAiso Plus (TaKaRa, Japan), and the cDNA was synthesized using HiScript II Q RT SuperMix for qPCR (Vazyme, Nanjing, China) according to the manufacturer’s instructions. *TaCNR2* sequences were cloned using the pair primers TaCNR2-F and TaCNR2-R with Phanta Max Super-Fidelity DNA Polymerase (Vazyme, Nanjing, China).

### Yeast tolerance

The open reading frame sequences of *TaCNR2* were amplified using the primers BXTaCNR2-F and BXTaCNR2-R, then constructed into plasmid pYES2 after digestion with *Bam*HI and *Xba*I. pYES2-*TaCNR2* and pYES2, the control as it was an empty vector, were transformed into *Saccharomyces cerevisiae* (BY4741) using the PEG/LiAC method (Invitrogen, USA), and cultured in solid uracil minus media at 30 °C for two days (FunGenome, Beijing, China). The concentration of all yeasts was modulated to an OD_600_ of 0.6, and further diluted to 10^0^, 10^−1^, 10^−2^, 10^−3^ and 10^−4^ with sterile H_2_O. Then, 4 μL of each dilution series were spotted onto solid media (yeast extract 2 g/mL; peptone 2 g/mL; galactose 20%) supplemented with 50 μM CdSO_4_, 5 mM ZnSO_4_ and 5 mM MnSO_4_. Growth was maintained for 3–7 days at 30 °C.

### Real-time quantitative PCR (RT-qPCR)

The wheat seedlings were exposed to 50 μM CdSO_4_, 200 μM ZnSO_4_ and 3 mM MnSO_4_ for 0, 3, 6, 12, 24 and 48 h. The shoots and roots were collected separately. An actin gene was used as an internal gene with the primer pair Taactin-F and Taactin-R. The RT-qPCR reaction was performed with 10 μL of HiScript II Q RT SuperMix for qPCR (Vazyme, Nanjing, China), 1 μL of cDNA, 0.5 μL each of the primers TaCNR2-FSSDL and TaCNR2-FSSDL (each 10 μM) and 8 μL ddH_2_O to a total volume of 20 μL. The transcript levels of *TaCNR2* in different wheat tissues and induced by different stressors were analyzed in a Roche LightCycler 480 Real-time System (Roche, Switzerland) and calculated using the 2^−ΔΔCT^ method^32^.

### Generation of overexpression in plants

*TaPCR2* was amplified with the primers TaCNR2-BFW and TaCNR2-xhRV, and constructed into plasmid pBI121 after digestion with *Bam*HI and *Xho*I. The plasmid was transformed into *Agrobacterium tumefaciens* GV3101 strain. The wild-type (WT) *Arabidopsis* (Col-0) was infected by transgenic *A. tumefaciens*, according to the floral dip method^33^. To generate transgenic rice, *TaCNR2* was amplified with the primers TaCNR2-1301-FW and TaCNR2-1301-RV, and cloned into plasmid pUN1301 using the Clone Express® II One Step Cloning Kit, according to the manufacturer’s instructions (Vazyme, Nanjing, China). The expression plasmid was transformed into *A. tumefaciens* GV3101. The experiments on transformed into rice (*Oryza sativa* L. *japonica* var Nipponbare) were performed at Hangzhou Biotech, Co., Ltd (Jiangsu, China).

### Tolerance of overexpressed *TaCNR2* plants

*TaCNR2*-transgenic *Arabidopsis* and rice seeds were sterilized with 75% ethanol and NaClO, and then washed five times with sterile water. To determine the root length and fresh weight, the *Arabidopsis* seeds were germinated on 1/2 Murashige and Skoog (MS) solid medium supplemented with 100 μM ZnSO_4_, 10 and 30 μM CdSO_4_, 1 and 3 mM MnSO_4_ and 2 mM H_2_O_2_ for 7–14 days, maintained at 23 ± 1 °C under an 8:16 h light: dark photoperiod. Root length and fresh weight were then measured. The rice seeds were germinated on 1/2 MS solid medium in the dark for three days at 37 °C. The control group was transferred into Kimura B solution for five days^34^ and maintained at 25 °C under a 16:8 h light/dark photoperiod. The treatment group was transferred into Kimura B containing 10 μM CdSO_4_, 100 μM ZnSO_4_ and 3 mM MnSO_4_ for 7–14 days. The length and fresh weight of the plants from both groups were measured after the experiment.

### Heavy metal content

*TaCNR2*-transgenic *Arabidopsis* and rice were incubated in 1/2 MS solid plate for seven and three days, respectively. After that, *Arabidopsis* was cultivated in liquid 1/2 HS for about 40 days, then transferred into media containing 30 μM CdSO_4_, 200 μM ZnSO_4_ and 3 mM MnSO_4_ for 2 days. Rice samples were cultured in Kimura B solution for 7 days, then supplemented with 10 μM CdSO_4_, 200 μM ZnSO_4_ and 3 mM MnSO_4_ for 7 days. The shoots and roots were collected separately. For the pot experiment, transgenic rice seedlings were cultivated in a greenhouse with 16 h light (28 °C)/8 h dark (25 °C) cycles using agricultural soil for four months. At the filling stage, rice plants were treated with mixtures of 5 mg/kg CdSO_4_/soil, 600 mg/kg ZnSO_4_/soil and 600 mg/kg MnSO_4_/soil for 40 days, respectively. Brown rice and the husks of mature rice plants were isolated and collected separately. The redundant metals on the surface of *Arabidopsis* and rice were eliminated after treatment with 10 mM EDTA for 30 min, then placed in an oven at 80 °C for four days. The dry weight of the collected plants was measured (mg) and subsequently digested in 8 mL of MOS level (HNO_3_) and 3 mL MOS level (H_2_O_2_) using microwave digestion (Milestone, Italy) for 60 min at 180 °C. The ion content of the digested samples was determined by inductively coupled plasma optical emission spectrometry (ICP-OES, Perkin Elmer, USA).

### Statistical analysis

All data are presented as mean ± standard error (SE) from three independent experiments. Statistical analysis was performed using the software programs Office 2010 and SPSS 13.0. The one-way ANOVA and *t*-test were used to compare the mean values (*p* < 0.05).
